# Effectiveness of expiratory technique and induced sputum in obtaining good quality sputum from patients acutely hospitalized with suspected lower respiratory tract infection: a statistical analysis plan for a randomized controlled trial

**DOI:** 10.1186/s13063-021-05639-1

**Published:** 2021-10-02

**Authors:** Mariana Bichuette Cartuliares, Helene Skjøt-Arkil, Flemming Schønning Rosenvinge, Christian Backer Mogensen, Thor Aage Skovsted, Andreas Kristian Pedersen

**Affiliations:** 1 Emergency Department, University Hospital of Southern Denmark, Kresten Philipsens vej 15, 6200 Aabenraa, Denmark; 2 Department of Regional Health Research, University of Southern Denmark, Aabenraa, Denmark; 3 Department of Biochemistry and Immunology, University Hospital of Southern Denmark, Kresten Philipsens vej 15, 6200 Aabenraa, Denmark; 4 Department of Clinical Microbiology, Odense University Hospital, J.B. Winsløwsvej 21-24, 5000 Odense C, Denmark; 5 Department of Research and Learning, University Hospital of Southern Denmark, Kresten Philipsens vej 15, 6200, Aabenraa, Denmark

**Keywords:** Randomized controlled trial, Statistical analysis plan, Sputum sample, Tracheal suction, Forced expiratory technique, Induced sputum, Respiratory tract infection

## Abstract

**Background:**

Targeted antimicrobial treatment is essential to avoid unnecessary use of broad-spectrum antibiotics and antimicrobial resistance. Targeted treatment relies on a precise microbiological diagnosis — in pneumonia, this poses a challenge as the usefulness of Gram stains and cultures is highly dependent on the quality of the sputum sample.

This study aims to examine adverse effects and quality of sputum samples obtained by expiratory techniques (forced expiratory technique and sputum induction) compared with tracheal suction. The hypothesis is that expiratory techniques are non-inferior to tracheal suction in obtaining samples from the lower respiratory tract. This statistical analysis plan (SAP) describes the study design, method, and data analysis of the trial to increase transparency, avoid reporting bias or data-driven analysis and increase the study’s reproducibility.

**Method:**

The design is a pragmatic, non-inferiority, parallel-arm randomized controlled trial including 280 patients admitted with suspected lower respiratory infection to two emergency departments. Patients are randomized to a usual care group, where sputum samples are collected by tracheal suction or to an intervention group where sputum samples are collected by forced expiratory technique and sputum induction. The statistical analysis will follow an intention-to-treat protocol. This SAP is developed and submitted before the end of recruitment, database closure, and statistical analyses.

**Discussion:**

The results of this study will provide valuable knowledge to clinical practice by comparing adverse effects and sputum sample quality associated with different sample methods.

**Trial registration:**

Clinicaltrials.gov, NCT04595526. Submitted on October 19, 2020

**Supplementary Information:**

The online version contains supplementary material available at 10.1186/s13063-021-05639-1.

## Administrative information {1}

Note: the numbers in curly brackets in this protocol refer to recommended guidelines for the content of a statistical analysis plan of a clinical trial developed by Gamble et al. [1] (Additional file 6). The protocol has been reported according to SPIRIT reporting guidelines [2].

**Table:** 

Full study title {1a}	Effectiveness of expiratory technique and induced sputum in obtaining good quality sputum from patients acutely hospitalized with suspected lower respiratory tract infection: a statistical analysis plan for a randomized controlled trial
**Clinicaltrials.gov****number {1b}**	NCT04595526 Registered on 10th October 2020
**Ethics Committee number**	S-20200133 Registered on 22th September 2020
**The Danish Data protecting Agency number**	20/41767 Registered on 11th September 2020
**SAP version {2}**	Version 1.0 date 18.12.2020
**Protocol version {3}**	Ethics Committee protocol version 4.0 date 26.01.2021
**SAP revision {4a}**	No SAP revisions. Protocol revised 1st of December 2020 Deviations from the protocol are described with justifications in section {19c}
**Roles and responsibility {5-}**	SAP protocol page 27 lines 537-542
**SAP author {6a}**	Mariana Bichuette Cartuliares, MSc, Ph.D. fellow Emergency Department, University Hospital of Southern Denmark, and Department of Regional Health Research, Faculty of Health Sciences, University Of Southern Denmark
**Statistician responsible {6b}**	Andreas Kristian Pedersen, cand.scient, Statistician Department of Research and Learning, University Hospital of Southern Denmark, and Department of Regional Health Research, Faculty of Health Sciences, University Of Southern Denmark
**Clinical lead {6c}**	Helene Skjøt-Arkil, M.Sc. Pharm., Ph.D., Associate professor Emergency Department, University Hospital of Southern Denmark, and Department of Regional Health Research, Faculty of Health Sciences, University Of Southern Denmark
**Collaborators/coming manuscript authors:**	Flemming Schønning Rosenvinge, MD Department of Clinical Microbiology, Odense University Hospital, Denmark Christian Backer Mogensen, MD, Ph.D., professor Emergency Department, University Hospital of Southern Denmark, and Department of Regional Health Research, Faculty of Health Sciences, University Of Southern Denmark Thor Aage Skovsted, MD, Ph.D. Department of Biochemistry and Immunology, University Hospital of Southern Denmark, Denmark

## Background {7}

Lower respiratory tract infection (LRTI) is a serious condition associated with high mortality, morbidity and economic burden [3–5]. Appropriate and targeted antimicrobial treatment is vital to avoid unnecessary use of broad-spectrum antibiotics and subsequent development of bacterial resistance.

Sputum Gram stain and culture from patients with LRTI are important when selecting a targeted antimicrobial treatment [6, 7].

Several clinical limitations interfere with the practice of obtaining a representative specimen from the lower respiratory tract (LRT). Many patients find it difficult to expectorate, sputum is often contaminated by oropharyngeal microbiota, and some patients have already been treated with antibiotics at admission, compromising the results from sputum culture [8–10]. The guidelines from the American Thoracic Society and Infectious Diseases Society of America recognize the challenges in obtaining a valid sputum sample. They recommend sputum analyses based on individual clinical assessment, local etiological considerations, and local antimicrobial stewardship [11].

The Danish guidelines [12] follow the National Institute for Health and Care Excellence guidelines [13] and are further developed by experts in the fields of microbiology, infectious disease, and emergency medicine. It is required that sputum samples must be collected at arrival at the emergency department (ED) from all patients admitted with suspected LRTI, and advocate tracheal suction as an optimal collecting method [12]. Despite indications from some studies that tracheal suction might be superior to expectorated sputa in diagnosing LRTI [14, 15], low accuracy and misclassification of poor samples have been reported [16, 17]. Tracheal suction is widely performed to clear pulmonary secretions in mechanically ventilated patients [18] but reported as a painful experience for the patient [19] being associated with adverse events such as hypoxia, oxygen desaturation, and mucosal bleeding [20]. Forced expiratory technique and sputum induction are considered safe and straightforward methods [21] and are used successfully to clear airways and increase sputum production, facilitating collection for microbiological analyses when patients are unable to expectorate [22–25].

The most effective method to collect a representative specimen from the LRT remains uncertain. The efficacy of tracheal suction, forced expiratory technique and sputum induction to obtain sputum samples of high quality has not been investigated in an ED context.

### Hypothesis, aim, and objectives {8 +12}

We hypothesize that expiratory techniques (forced expiratory technique and sputum induction) are non-inferior to tracheal suction in obtaining sputum samples from the lower respiratory tract.

This study aims to investigate the effectiveness of expiratory techniques compared to tracheal suction on the quality of sputum samples collected from patients acutely hospitalized with suspected LRTI.

The following objectives will be explored:

1. Is the proportion of suitable (good quality) sputum samples different when comparing samples obtained by tracheal suction to samples obtained by expiratory techniques?
2. Is the number of adverse events different when comparing tracheal suction to expiratory techniques?
3. How do patients experience the two sputum sample collecting procedures?

This statistical analysis plan (SAP) will describe the statistical analysis to be executed on primary and secondary outcomes to increase study transparency, reproducibility, and validity. This SAP was developed after the study protocol was registered with the following organizations: Danish ethics committee; clinical trials; data protection agency and, before database closure, data review; and commencement of any statistical analyses.

## Method

This SAP followed the recommended guidelines for a statistical analysis plan of a clinical trial developed by Gamble et al. [1]. A checklist for SPIRIT reporting guidelines and a template for the schedule of enrolment [2] is attached as Additional files 1 and 3. The study will follow the Consolidation Standard of Reporting Trials (CONSORT) guidelines (parallel-group randomized trials) [26].

### Study design and setting {9}

The study design is a pragmatic, non-inferiority, randomized controlled trial (RCT). The trial is conducted from the 9th of November 2020 until the 1st of July 2021 at the University Hospital of Southern Denmark including the hospitals of Aabenraa and Sønderborg with a hospital coverage of approximately 250,000 inhabitants. The standard method for sputum collection at both EDs is tracheal suction.

Six project assistants from the ED (experienced nurses and physiotherapist) will identify eligible patients and collect informed consent (Additional file 5) and data. The project assistants will receive bedside and simulation training in all the included sample methods on how to obtain an optimal specimen. Furthermore, a guideline on how to perform the expiratory technique will be developed to support consistent data collection.

### Trial population

#### *Screening data* {21}

Screenings data from previous studies was used to estimate the sample size of our trial population [9, 10, 23, 27, 28]. Use of this data ensures our results will be reproducible.

#### Eligibility {22}

**Fig. 1:**
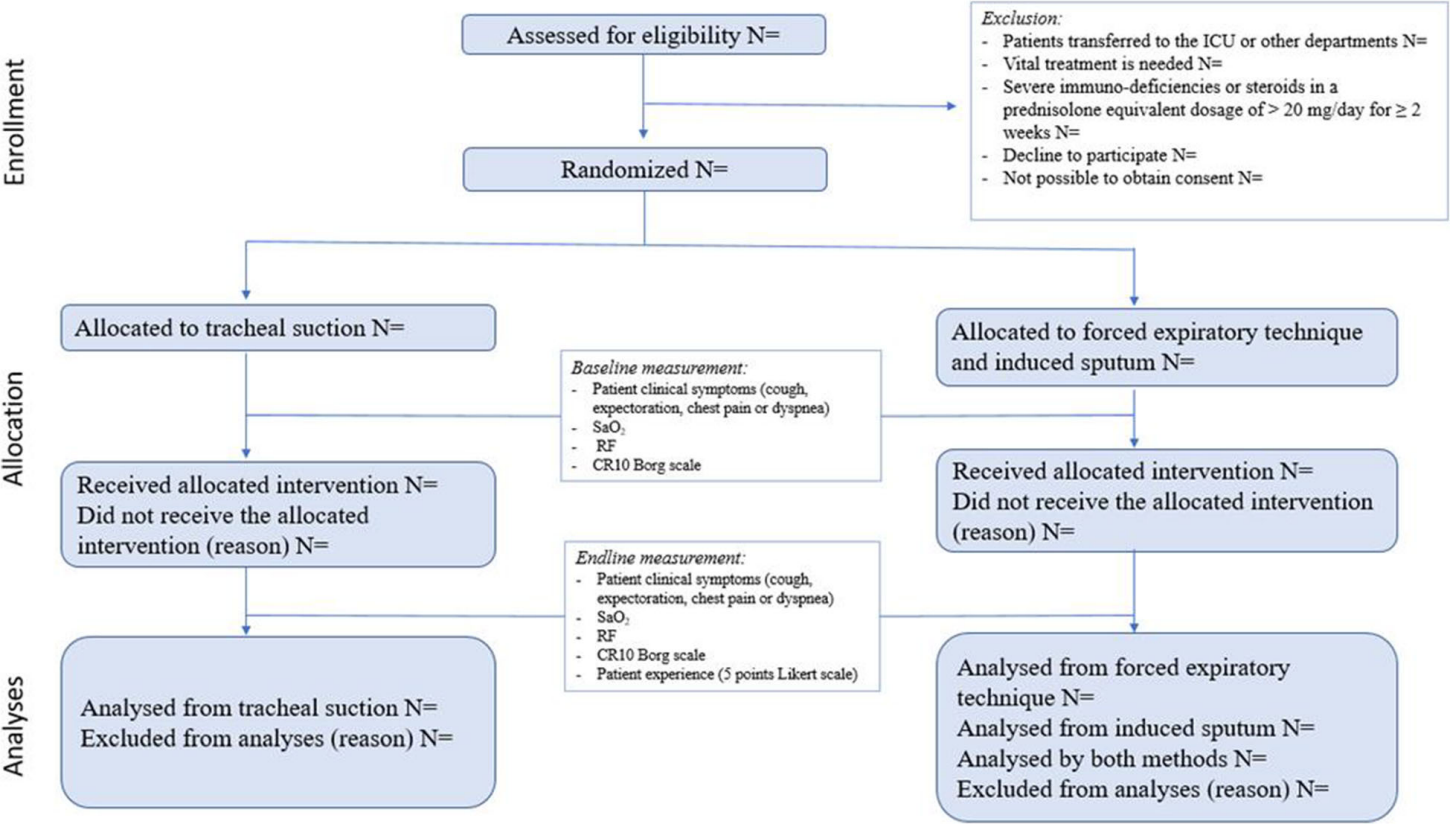
Recruitment procedure and allocation

Adults admitted to the ED with suspected LRTI will be invited to participate in the study if the attending physician identifies one of the following pulmonary symptoms: dyspnea, cough, expectoration, chest pain, or fever (Fig. 1).

Patients will be excluded if the following occur:

- The attending physician finds that participation will delay urgent treatment
- Patients are transferred to an intensive care unit
- Patients have severe immunodeficiencies such as human immunodeficiency virus infection (a CD4 count below 200/μl)
- Patients are treated with immunosuppressive therapy such as chemo- or radiotherapy
- Patients are treated with steroids in a prednisolone equivalent dosage of more than 20 mg/day for 2 weeks up to the current admission or if consent cannot be obtained

#### Recruitment {23}

Admitted patients with suspected LRTI will be identified in the patient management system (CETREA 4.2.0.0.) at the ED by a project assistant. The attending physician will confirm eligibility, and the patient’s verbal and written consent will be obtained by the project assistant within the first hour of admission.

### Randomization {10}

Within an hour after recruitment, enrolled patients are randomly assigned to either control (sputum collection by tracheal suction) or intervention (sputum collection by forced expiratory techniques). Patients in the intervention group who cannot deliver a specimen will undergo tracheal suction according to regional guidelines, and these samples will not be included in the primary analyses.

Patients are allocated 1:1 according to a computer-generated randomization sequence without stratification using blocks of six programmed by an independent data manager, so the treatment assignment is made by chance and the allocation is completely independent of any prior treatment allocation to a patient. The randomization tool in Research Electronic Data Capture (REDCap) will be used [29, 30]. The project assistants administering the randomization will not have access to the randomization code and will be blinded to the randomization procedure, block sizes, and randomization sequence at all times during the trial period. In addition, allocation concealment is ensured, as the randomization is performed electronically, is not revealed before consent is obtained, and occurs immediately prior to sputum collection.

#### Blinding

Participants, project assistants, and outcome adjudicators are not blinded to the randomization as it is not possible. The investigator will be blinded until data analysis is completed.

### Exposure variables

#### Tracheal suction

Before tracheal suction is performed, the patient will be informed in detail about the procedure. The patient is introduced to Fowler’s position (semi-sitting position in 45–60° with knees either bent or straight) and encouraged to clear their airway with a deep cough. The catheter (EXTRUDAN Surgery Aps, Denmark, CH12, 530 mm) tip is lubricated with Xylocaine (lidocaine HCl) 2% jelly and inserted into the nares during inhalation and gently advanced about 40 cm without applying suction. The suction catheter port is covered, and suctioning is performed before withdrawing the catheter. Suction is set at 200–400 mmHg negative pressure. If necessary, the specimen is diluted in sterile saline water before storage in a sterile container.

#### Sputum samples collected by forced expiratory techniques

These techniques are based on the patients’ own attempts to deliver a sputum sample. The patient is brought into a 90° sitting position and instructed to clear the mouth with water to minimize oropharyngeal contamination, and proper forced exhalation and the coughing technique are thoroughly explained [24, 31].

The patient takes 3 to 5 deep diaphragmatic breaths, inhaling through the nose, exhaling through pursed lips. The patient takes a normal breath and then squeezes it out by contracting the abdominal and chest wall muscles, with the mouth and glottis open, while whispering and forcing the word “huff” during exhalation. The procedure is repeated 3 to 5 times. As secretions enter the larger airways, the patient exhales from high-to-mid lung volume to clear secretions from more proximal airways. The procedure continues as the patient takes 3 to 5 relaxed diaphragmatic breaths before coughing again [24, 31]. The sputum sample is stored in a sterile container.

#### Induced sputum

Isotonic saline inhalation (0, 9%) is used to loosen and induce the sputum. It is given for 10 min with a nebulizer system (Unomedical Opti-Mist TM,2.1m, ref. 93-772mm) [22]. After that, a forced expiratory technique is performed, and the sputum sample is stored in a sterile container.

Inhalation medicine is permitted as part of concomitant care.

### Microbiological variables

#### Microscopy

Part of the sputum is placed on a microscope slide with a cotton swab, and a second microscope slide is used to distribute the material on the surface. The smear is then heat-fixed and Gram stained. The number of squamous epithelial cells and polymorphonuclear leucocytes per field of view (100× magnification) is registered. Samples with < 10 squamous epithelial cells per field of view are classified as suitable (good quality), samples with ≥ 10 squamous epithelial cells are classified as unsuitable (poor quality) [32]. The microscopy findings and classification are registered in the microbiological laboratory information system (MADS, Aarhus University Hospital, Aarhus, Denmark) and are accessible in the patient’s file.

In addition, a different sputum quality assessment based on both squamous epithelial cells and polymorphonuclear leucocytes will be used in sub-analyses. Samples with < 10 squamous epithelial cells and ≥ 25 polymorphonuclear leucocytes per field of view (100× magnification) are classified as suitable (good quality) [32].

The samples unable to be collected will be considered missing as the quality cannot be determined.

#### Culture

Sputum is transferred with a cotton swab to an agar plate with 5% sheep blood (Beckton Dickinson) with a *Staphylococcus aureus* streak and a Chrom Orientation agar plate (Beckton Dickinson), and the inoculum is streaked over the agar surface. The blood agar is incubated at 35 °C in an atmosphere with 6% CO_2_, and the Chrom-agar orientation is incubated at 35 °C in normal atmospheric conditions.

After 1–2 days of incubation, pathogens are identified and tested for antimicrobial susceptibility.

The pathogen identified by culture will be semi-quantitatively assessed as numerous, some, or few, with a morphology description. Mixed flora judged to be pharyngeal flora will be registered as “pharyngeal flora”. No growth of pathogens will be registered as “No growth of pathogens”.

### Baseline variables {25}

Demographics, symptoms, and smoking are collected from a patient interview. Vital parameters, comorbidities, suspicion of pneumonia, diagnostic packages [33], severe acute respiratory syndrome coronavirus 2 (SARS-CoV-2), disease severity assessment (CURB-65 [34], PSI [35], and Danish Emergency Process Triage (DEPT) [36]), blood tests (C-reactive protein, leucocytes, neutrophils) length of hospital stay, antibiotic consumption, and inhaled medicine are extracted from the patient’s medical record (Table 1).

**Table 1:** Demographic and baseline clinical characteristics

Variable	Overall	Group 1 total (*n* =)	Group 2 total (*n* =)
		Tracheal suction (*n* =)	Forced expiratory technique (*n* =), induced sputum (n =)
Site			
Aabenraa			
Sønderborg			
Sex, *n* (%)			
Age, mean (SD)			
Smoking status			
Current smokers, *n* (%)			
Ex-smokers, *n* (%)			
Non-smokers, *n* (%)			
Symptoms			
Cough, *n* (%)			
Expectoration, *n* (%)			
Chest tightness, *n* (%)			
Dyspnea, *n* (%)			
Severity assessment			
CURB-65 score* 0–1, *n* (%)			
CURB-65 score* 2, *n* (%)			
CURB-65 score* 3–5, *n* (%)			
PSI* risk class 2–3, *n* (%)			
PSI* risk class 4, *n* (%)			
PSI* risk class 5, n (%)			
Triage level 1, *n* (%)			
Triage level 2–3, *n* (%)			
Triage level 4–5, *n* (%)			
Diagnostic packages			
Airways symptoms, *n* (%)			
Fever, *n* (%)			
Dyspnea, *n* (%)			
Unspecific illness, *n* (%)			
Suspicion of COVID-19 infection, *n* (%)			
Other, *n* (%)			
Suspicion of pneumonia, *n* (%)			
SARS-COV-2 positive at admission, *n* (%)			
Comorbidities			
COPD*, *n* (%)			
Asthma, *n* (%)			
DM*, *n* (%)			
CVD*, *n* (%)			
Cancer, *n* (%)			
Other, *n* (%)			
Vital parameters			
SaO_2_*, mean (SD)			
RR*, median (IQR)			
HR*, median (IQR)			
Systolic blood pressure, median (IQR)			
Diastolic blood pressure, median (IQR)			
Fever > 38 °C, *n* (%)			
Altered mental status, *n* (%)			
Blood tests			
C-reactive protein, mean (SD)			
Leucocytes, mean (SD)			
Neutrophils, mean (SD)			
Did the patient receive antibiotic treatment			
Within 1 month, *n* (%)			
Before sputum collection, *n* (%)			
Inhaled medications, *n* (%)			
Length of hospital stay, mean (SD)			

*CURB-65 score, score for pneumonia severity based on confusion, urin, respiratory rate, blood pressure, and age; *PSI*, Pneumonia Severity Index; *COPD*, chronic obstructive pulmonary disease, *DM*, diabetes mellitus type 1 and type 2; *CVD*, cardiovascular disease; *SaO*_*2*_, oxygen saturation; *RR*, respiratory rate; *HR*, heart rate

### Co-variables

Co-variables (listed and defined below) will be measured and compared between the two allocation groups. Patients’ symptoms, oxygen saturation, respiratory rate, and overall experience will be measured before (right after allocation) and after sputum collection (measured at the latest 10 min after the procedure). Side effects observed by the project assistants and experience by the patient during the procedure will be registered once after sputum collection. Data on mortality and re-admission will be extracted from the patient medical record. The projects assistants will follow standardized protocols to register all the co-variables including the questions concerning patient experiences.

- C*oughing, expectoration, dyspnea, and chest pain* symptoms will be reported as binary variables. Aggravation of cough, expectoration, dyspnea, and chest pain experienced by the patient will be reported.
- *Oxygen saturation.* Oxygen saturation (SaO_2_) will be measured using a pulse oximetry device to measure arterial oxygen saturation level in percentages up to 100%. The acceptable SaO_2_ is ≥ 93%. For patients with chronic obstructive disease, a SaO_2_ is acceptable at ≥ 88%. SaO_2_ will be reported as mean (standard deviation (SD)) [37].
- *Respiratory rate.* The respiratory rate (RR) will be counted by the professional attending the patient. The respiratory rate is the number of breaths per minute that a patient takes while resting. It is assessed by counting the number of times the patient’s chest rises in half a minute multiplied by two. RR will be reported as mean with standard deviation (SD). Acceptable results for RR are between 12 and 20 times per minute [38].
- *Side effects observed by the project assistants.* Project assistants will register any side effects such as bleeding from the airways and bronchospasm.
- *Short-term mortality.* Mortality within 7 days from admission to the emergency department
- *Re-admission.* Re-admission to hospital within 30 days of the original discharge
- *Patient’s overall experience of symptoms.* Patient wellbeing and overall experience of symptoms will be measured at the latest 10 min after sputum collection. Patients will asked to verbally score their overall experience of their symptoms based on the CR10 Borg scale [39, 40]. The CR10 Borg scale assess the experience of patient symptoms ranges from “0= nothing at all” to “10 very very strong” and over 10 as “maximal” [40]. A visual support tool describing this scoring system will be used to assist the patient and the ordinal result will be reported as mean and SD.
- *Patient’s experience of the procedure to collect sputum sample*: This will be measured once after an attempt at sputum collection. Patients will be asked to give a verbal score to the question: “What was your experience of this procedure?” using a five-point Likert scale. This scale ranges from “very bad, bad, neither bad nor good, good, very good”. A visual support tool describing this scoring system will be used to assist the patient. The result will be reported in percentages. The development and validation of this scale was based on individual and focus group interviews using cognitive interview methods [41].

### Outcome definitions {26}

1. *Primary outcome*. The quality of sputum samples, binary outcome
2. *Secondary outcome 1*. Number of adverse events, discrete outcome
  - Aggravation of SaO_2_ and SaO_2_ ≤ 93% (COPD patients ≤ 88%), binary
  - Aggravation of RR and RR lower than 12 or higher than 20 times per minute, binary
  - Occurrence or aggravation of symptoms (cough, expectoration, dyspnea, chest pain), binary
  - Aggravation of patient symptoms experience, binary
  - Occurrence of side effects observed by the project assistant, binary
  - Death within 7 days from admission, binary
  - 30 days re-admission after current hospitalization, binary
3. *Secondary outcome 2*. Patient experience of the sputum collection procedure, ordinal outcome

### Harms {30}

The project includes collecting methods that are already applied and recommended in clinical practice. There are minimal indications of adverse effects of a spontaneous cough or induced sputum after inhaling an isotonic saline solution (0.9%) [21, 22]. It is known that the tracheal suction routine carries a risk of airway bleeding, although this is rare and often short-lived [20]. Moreover, there may be short-term discomfort, especially during the tracheal suction procedure [19]. Patients will be informed of the adverse events verbally and in writing. In case of an aggravation of symptoms, the procedure that is being performed will be interrupted preventing patient participation. The procedures are known to have minimal risks and the hospital will provide routine ancillary post-trial care, if necessary.

### Data management {32}

Data management plan version 1.0 is attached to the Additional file 4.

### Data monitoring

During the data collection, an extern assessor will supervise the performance of all project assistants and an independent microbiologist expert will ensure data quality and continue registration of the specimens. The daily inclusion of participants will be monitored by the project investigator and discussed with the study assistants and steering committee.

After primary analysis of data, the results will be discussed and evaluated first in the steering committee and afterwards with all involved hospital departments.

## Statistical method

### Sample size consideration {11}

It is hypothesized that the intervention group receiving expiratory techniques is non-inferior to the tracheal suction group. Grouping two methods in the intervention arm, there will be fewer missing values and only two comparison groups.

Based on clinical practice and literature, it is assumed that no sample will be obtained from 50% of patients by forced expiratory technique alone [9, 10, 42], 30% from patients by sputum induction combined with the forced expiratory technique [23, 43], and 10% from patients using tracheal suction [28]. The proportion of obtained samples classified as good quality is assumed to be 75% for the procedures using expiratory techniques [23, 27, 43] and 90% for tracheal suction [28].

Considering these assumptions, the study’s power is 84% with 260 patients, including missing values. The power of the study was calculated with a Monte Carlo simulation using logistic regression.

An internal register showed that at least 1250 patients were admitted to the study ED with suspicion of infection annually, and of these, 600 patients were diagnosed with pneumonia. Including 260 patients with suspected LRTI will take five months, taking into account exclusion criteria, weekends/holidays, and missing data.

To avoid overfitting in the primary analysis, we followed the relaxed one in ten rule for the fixed effects and one in 20 rule for the random effects [44].

### Statistics principles

#### Statistical interim analyses and stopping guidance {13}

No interim analysis will be conducted.

#### Timing of final analyses {14+15}

The final analyses will be conducted within three months of completion of inclusion, estimated to be ultimo September 2021. All outcomes will be analyzed collectively.

#### Confidence interval and *p*-value {16+17+18}

For the main analysis and the supplementary analysis with non-ordinal outcomes, 95% confidence intervals will be reported. For the supplementary analysis of the secondary outcomes, bootstrapped confidence intervals will be calculated for ordinal outcomes to accommodate lack of fit. A *p*-value less than 0.05 will be considered statistically significant.

No adjusting for multiple testing will be utilized in any analyses, as we only have one primary outcome [45].

#### Definition of analyses population {20}

The primary analysis will be conducted according to the intention-to-treat (ITT) principle [46, 47].

We define the ITT population as all admitted patients suspected of LRTI who sign informed consent and subsequently are allocate to one of two sputum collection groups. Sputum samples obtained by tracheal suction from patients in the intervention group, who are not able to expectorate, will be analyzed separately from the ITT analyses.

The primary analysis compares the tracheal suction group and the expiratory technique group to identify the most effective method for obtaining good quality sputum samples. The secondary analysis consists of an agreement analysis [48] between forced expiratory technique alone and forced expiratory technique after sputum induction.

Additionally, analysis of the secondary outcome of adverse effects will be conducted at baseline (right after allocation) and at the latest, 10 min. after the intervention. Clinical symptoms (cough, expectoration, chest pain, or dyspnea), SaO_2_, RR, and CR10 Borg scale [40] will be measured. Furthermore, a 5-point Likert scale will be used to measure patient experience of the procedure. The analyses will be adjusted for variables with a large influence on the outcome, which is an odds ratio larger than 2, to accommodate bias [49].

Descriptive analyses will be performed on the pathogens found when the specimens are cultured according to differential quality criteria.

### Statistical analysis {27}

#### Descriptive statistics

Descriptive statistics will be utilized to assess if exchangeability is fulfilled for baseline variables. For categorical variables, Fischer’s exact test or chi-square test will be used to test if the distribution between the two groups is different. For discrete and continuous variables, the Wilcoxon rank-sum test or Student *t*-test will be utilized to assess if the distribution is similar between the two groups depending on normal data distribution.

#### Analysis for primary outcome

For the primary outcome, the following analyses will be utilized:

1. To assess the suitability of the sputum sample between the two collecting methods groups, a hierarchical mixed effect logistic model with and without imputed values will be utilized to accommodate the random effect’s hierarchical structure, which manifests according to different personnel collecting the samples and geographical variation (Table 2).

**Table 2:** Results of the primary outcome regarding specimens’ suitability and secondary outcome of adverse effects and patient experience. Tracheal suction method (reference group) compared to expiratory technique methods

Outcome	Unadjusted OR (CI) ***P***	Adjusted OR (CI) ***P***
**Quality assessment of the specimens**		
**Adverse effects**		
	**Unadjusted Coef (CI)*P***	**Adjusted Coef (CI)*P***
**Patient experience**		
Five-point Likert scale
2. Sub-analyses
  - To assess agreement between forced expiratory technique alone and induced sputum combined with the forced expiratory technique, a kappa statistic will be calculated [48].
  - If the kappa statistic indicates a lack of agreement between forced expiratory techniques [50], a sensitivity analysis comparing forced expiratory technique with tracheal suction and induced sputum with tracheal suction will be utilized. A hierarchical mixed effect logistic model will be utilized for both analyses.
  - For patients where expectorate cannot be obtained in the intervention group, descriptive analyses will be conducted on the numbers of tracheal suctions performed and the obtained specimen quality.
  - Kappa statistics will be performed to identify differential quality criteria of Gram stain results classified in three groups (Table 3). Bacterial pathogens are described in Table 4.

**Table 3:** Gram stain results classified according to the number of epithelial cells and polymorphonuclear leucocytes judged by microscopy (100× magnification)

Epithelial cells	Polymorphonuclear leucocytes		
	< 10	10–24	≥ 25
≥ 25			
10–24			
< 10

**Table 4:** Bacterial pathogens identified in culture according to differential quality criteria. Results are presented in percentages

	Squamous cells < 10	Squamous cells 10–24	Squamous cells ≥ 25
**Bacterial agents**			
*Streptococcus pneumonia*			
*Haemophilus influenza*			
*Moraxella catarrhalis*			
*Pseudomonas aeruginosa*			
*Staphylococcus aureus*			
*Enterobacteriaceae*			
*Hemolytic streptococci*			
Pharyngeal flora			
No growth of pathogens			
Others

#### Analysis for secondary outcomes

For the secondary outcomes regarding adverse events and patient experience, the following analyses will be utilized (Table 2):

1. To investigate if the number of adverse events differs between groups, a mixed effect Poisson model will be utilized with and without imputed values to accommodate the random effect’s hierarchical structure, which manifests according to different personnel collecting the samples and geographical variation.
  - A sensitivity analysis for each adverse event type by either a chi-square test or Fischer’s exact test will be performed (Table 5).

**Table 5:** Sensitivity analyses for adverse events

Adverse events	Expiratory techniques, ***n*** (%)	Tracheal suction, ***n*** (%)	***P***
**Vital parameters**			
SaO_2_^a^			
RR^a^			
**CR10 Borg scale**			
**Side effects**			
Bleeding			
Bronchospasm			
Others			
**Patient symptoms**			
Cough			
Dyspnea			
Expectoration			
Chest tightness			

^**a**^*SaO*_*2*_, oxygen saturation; *RR*, respiratory rate
2. For the procedure experience using a five-point Likert scale, a Wilcoxon rank test will be performed to compare the two allocation groups according to sample methods.

Model control for the generalized linear mixed models will consist of assessing the deviance residuals’ normality assumptions for each center by way of quantile-quantile plots. To assess if the variables in the descriptive analysis are normally distributed, quantile-quantile plots will be utilized.

If any of the generalized linear mixed models do not converge, a simplification of the covariance structure will be utilized.

### Withdrawal from the study {24}

It is assumed that there will be minimal patient withdrawal from the study, as the specimens will be collected immediately after patient consent and allocation, and there will be no further patient contact. Withdrawals will be assumed to be missing completely at random (MCAR) [51].

### Missing data {28+29}

Missing data includes patient withdrawals and sputum samples that are not possible to collect due to patients’ nonproductive cough or noncompliance. Multiple imputations and complete case analysis will be conducted to investigate the influence of missing data on the results [52]. The guidelines concerning missing data suggested by Sterne et al. will be followed to ensure transparency [53].

### Statistical software {31}

Statistical analyses will be performed using STATA version 17.0.

## Planned figures and tables

The flow of participants will be illustrated in a flow diagram according to the CONSORT guidelines [54].

Baseline characteristics will be summarized in Table 1. Data will be presented as means with SD when normally distributed or as a median with interquartile range if the data is skewed. Dichotomous and categorical variables will be presented with number and percentage.

## Discussion

The results of this study will provide important evidence on the optimal routine for obtaining high-quality sputum samples. This knowledge is essential when aiming to improve the initial management of patients admitted with suspected LRTI by supporting clinical decisions and targeted treatment.

The study will run in a real-life setting to increase the feasibility in implementing the methods afterwards.

This pre-defined SAP is essential to increase the study’s transparency and explicitly describe protocol deviations to increase reproducibility, avoid any risk of reporting bias or data-driven analysis.

## Adherence and protocol deviations {19}

### Adjustments after the study start and before recruitment completion

Due to the SARS-CoV-2 pandemic, patient distribution was altered, and it was necessary to extend the recruitment to include the second site. The study period was adjusted with study start from October 19th to November 9th, and study completion from March 1st to July 5th and will include at least 260 participants. New sample size and power calculations were performed where the clinical significant absolute difference was set at 15% points. The personnel conducting the sample size calculations do not know the participants’ outcomes.

### Adjustments before study start but after study protocol acceptance

The increased SARS-CoV-2 situation did not make it possible to investigate the washed sputum technique analyses as it requires training of the staff. Therefore, this analysis was withdrawn from the study. The quality criteria for all randomized patients will follow the standard used by the microbiological department to reduce missing data (sputum samples containing < 10 squamous epithelial cells per low power field of view are registered as usable material). The quality criteria of squamous epithelial cell and polymorphonuclear leucocytes classified in groups of < 10, 10–24, ≥ 25 per low power field of view, will be registered as intended when available, making possible secondary analyses on sputum suitability.

All patients included in the trial will be asked how they experienced the collected method based on a five-point Likert scale instrument developed before the study start.

Protocol amendments will be registered directly with ClinicalTrials.gov (NCT04595526) (Additional file 2) and ethics committees and described explicitly in future publications.

## Supplementary Information

**Additional file 1.** Spirit schedule.

**Additional file 2.** World Health Organization trial registration data.

**Additional file 3.** Spirit checklist.

**Additional file 4.** Data management plan.

**Additional file 5.** Informed consent.

**Additional file 6.** SAP guideline.

## Data Availability

Due to Danish laws on personal data, data cannot be shared publicly. The person responsible for the research is the principal investigator and corresponding author that together with the Department of Health Research and the University Hospital of Southern Denmark owns the data and has access to the final data-set. To request this data, please contact the corresponding author for more information. For ancillary studies, a new consent will need to be given by the Regional Committees on Health Research Ethics for Southern Denmark.

## Abbreviations

ED: Emergency department
LRT: Lower respiratory tract
LRTI: Lower respiratory tract infection
OPEN: Open Patient data Explorative Network
REDCap: Research Electronic Data Capture
SAP: Statistical analyses plan
