# Supplementary figures and images for: Effectiveness of expiratory technique and induced sputum in obtaining good quality sputum from patients acutely hospitalized with suspected lower respiratory tract infection: a statistical analysis plan for a randomized controlled trial
Source: Trials. 2021 Oct 2;22:675. doi: 10.1186/s13063-021-05639-1 (PMC8487344; doi:10.1186/s13063-021-05639-1)

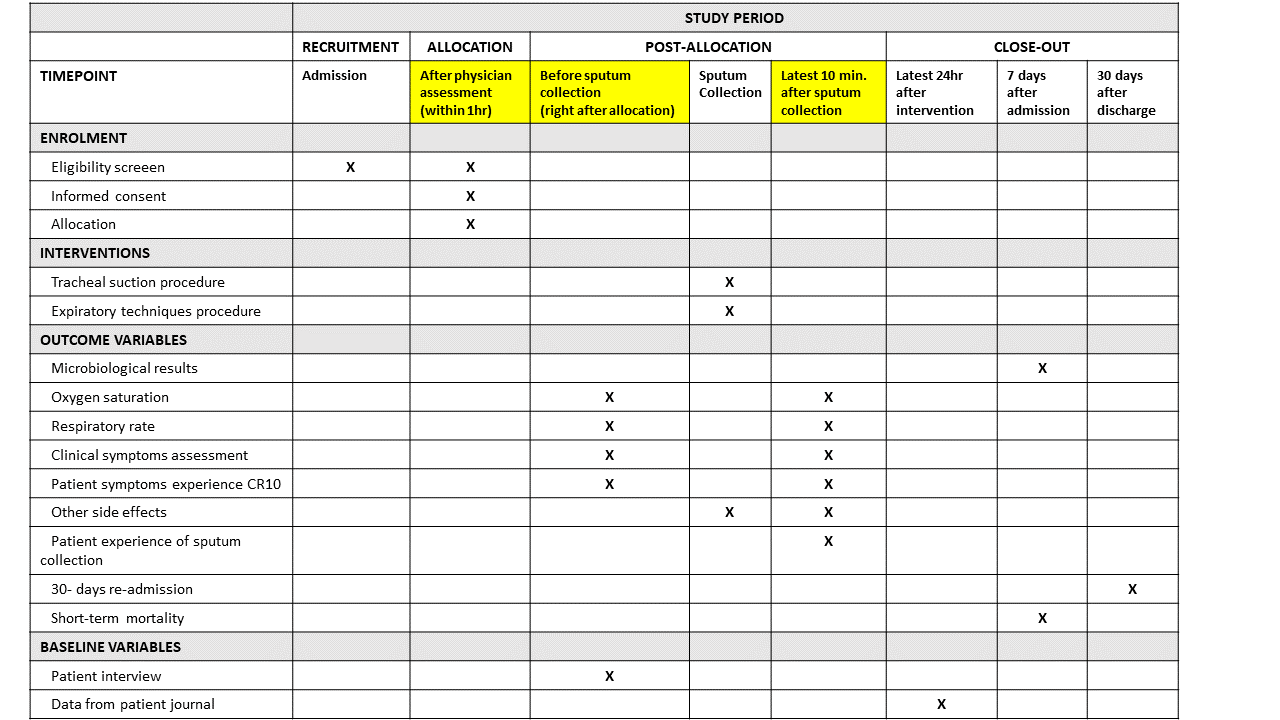

Supplement: Supplementary file 1 — Additional file 1. Spirit schedule. [file 13063_2021_5639_MOESM1_ESM.png]

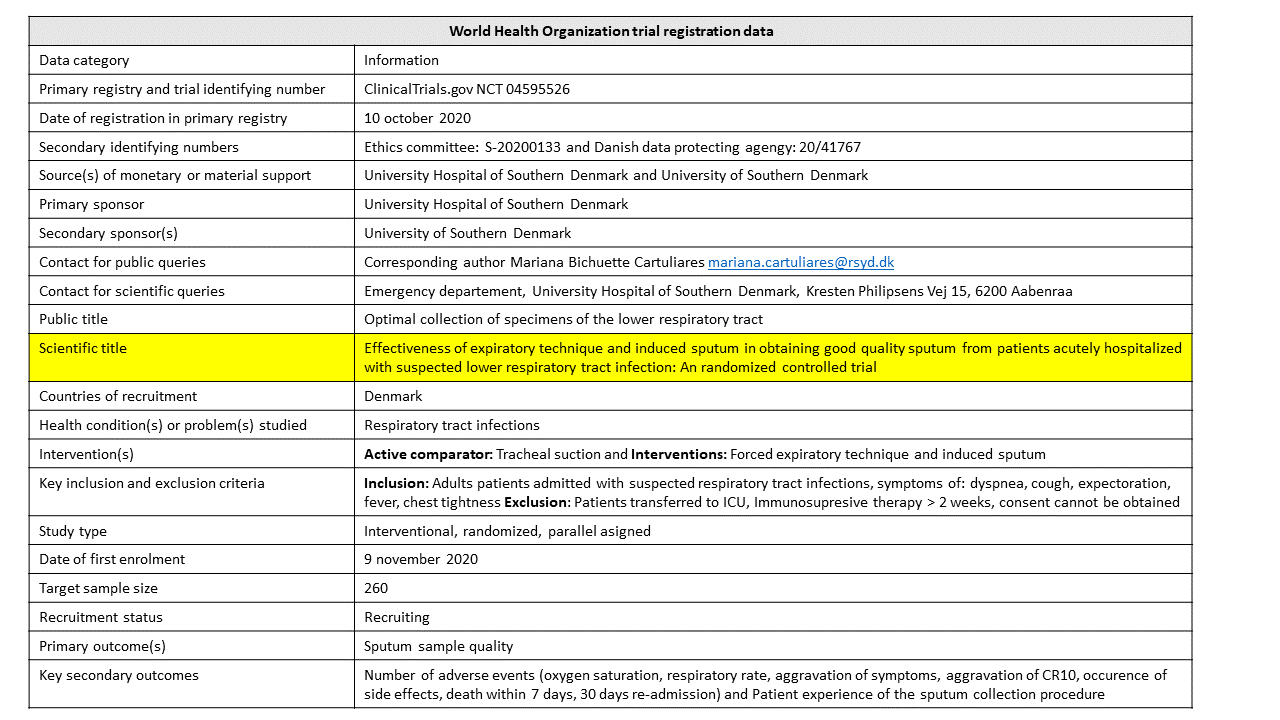

Supplement: Supplementary file 2 — Additional file 2. World Health Organization trial registration data. [file 13063_2021_5639_MOESM2_ESM.png]
