# Supplementary material for: Effectiveness of expiratory technique and induced sputum in obtaining good quality sputum from patients acutely hospitalized with suspected lower respiratory tract infection: a statistical analysis plan for a randomized controlled trial
Source: Trials. 2021 Oct 2;22:675. doi: 10.1186/s13063-021-05639-1 (PMC8487344; doi:10.1186/s13063-021-05639-1)
Supplement: Supplementary file 4 — Additional file 4. Data management plan. [file 13063_2021_5639_MOESM4_ESM.pdf]

Data management plan

Effectiveness of expiratory technique and induced sputum in obtaining good quality sputum from patients acutely hospitalized with suspected lower respiratory tract infection

Data management template used

https://dmponline.deic.dk/

Data Collection

The data is collected from the Hospital of Southern Denmark and include the Emergency Department in Aabenraa and Sønderborg.

Type of data:

- Patient interview

Patient interview will be performed in conjunction to the collection of sputum samples. It includes patient's smoking status, vital signs, patient's symptoms before and after sputum sample collection (adverse events), and experience of the procedure.

- Medical record

The data extracted from the medical record will comprise diagnosis, comorbidities, blood test results, sputum analysis, chest x-ray, disease severity assessment, inhaled medicine, severe acute respiratory syndrome coronavirus 2 (SARS-CoV-2), length of stay, readmission, mortality, antibiotic treatment prescribed within last month and upon current admission. All data will first be collected after written consent has been obtained from the patients.

- Data from the Department of Microbiology

Data from the Department of Microbiology related to Gram stain and culture results will be extracted from the internal system of the microbiological laboratory information system (MADS, Aarhus University Hospital, Aarhus, Denmark)

Format and volume:

The data is collected and stored using REDCap (Research Electronic Data Capture) that is a electronic data capture tools hosted at OPEN (Open Patient data Explorative Network, Odense University Hospital, Region of Southern Denmark)- University of Southern Denmark and is a secure, web-based software platform designed to support data capture for research studies.

Excel will be used in extraction of the data from the department of microbiology. Excel can be merged with data from REDCap. STATA and R will be used in data analyses. Both formats, Excel, STATA and R fils will be stored in the regional close secure system in Sharepoint or hospital logged drev.

The volume of the data will not exced the space available.

Data structure

Data will be structured by the following folders:

- Patient inclusion (identifying whether the patient is included or not in the study)
- Patient interview
- Data extracted from patient medical record
- Clinical endpoints (data regarding to the study main outcome including microbiology Gram stain and culture, length of stay, readmission and mortality)

All files will be date signed by year, month and day, to secure that the last version will be used.

Guidelines from OPEN, University of Suthern Denmark will be used to cler and handle the data:

https://www.sdu.dk//media/files/om_sdu/institutter/klinsk+institut/forskningsenheder/open/vejledninger/guide+til+registerforskning+ny.pdf

Quality assurance of the data collected

The data extracted from the patient medical record will be revised by two project assisstants before locked by the project investigator.

Sputum sent to Gram stain and culture are supervised by the chief microbiologist at the arrival to the microbiologist department and after Gram stain and culture.

Data management is supervised by an independent datamanager from OPEN, University of Suthern Denmark, assuring quality and data security.

Documentation and metadata

Documentation of the data

The data based on the patient interview and the patient medical record will be captured in REDCap (Research Electronic Data Capture), University of Southern Denmark.

A protocol with instructions on sputum collection is used to increase the consistency in data collection. Questions to the patient under patient interview are standardised and structured to minimize intervariability between data collectors. During the data collection, an extern assessor will supervise the performance of all project assistants.

Variables from the patient interview and from the medical record are specific and predifined by the measured unit used e.g. for blood pressure (mmHg) or oxygen saturation measured by a pulse oximetry device measured en percent (%). Furthermore, the place where the information must be extracted from is described in conjunction to the variable e.g. time of antibiotic consumption before sputum collection (patient medical record-admistration list of medicine)

Outcome measures regarding primary and secondary outcome are described in detail in the protocol to the ethics committe (S-20200133). Metadata and the protocol wil not be available online but it will be possible to require it by sending an e-mail to the responsible for the study registered in clinicaltrials.gov (NCT04595526) or by corresponding to the corresponding author in the future publication.

The analyses will be performed in STATA in the secured system REDCap and a master do-file will be developed to describe all do-files used in the analyses process.

Results that will be published will be completely anonymized patient-level dataset and corresponding statistical code will be made publicly available if required by the scientific journal, in which the results are published.

The data will be stored and will be eliminated by the end of 2027 according to data protecting agengy number (20/41767).

Ethics and legal compliance

Writing consent is obtained from all participants regarding data collection, inclusion in the study and anomized results. Sensitive data as personal security number and name are immediately deleted and converted to an ID number. Data will be pseunomized during data collection and analysis.

Participants can any time regret the consent and all data will be deleted.

Written consent is stored in a locked safety room.

All necessarily approvals to the project will be reported following the guidelines:

https://www.sdu.dk/da/om_sdu/institutter_centre/klinisk_institut/forskning/forskningsenheder/open/nyttige_links

The data will be preserved according danish law and regulations and will not be shared to third-party. The study is accepted by the ethichal committe (S-20200133).

Data owner

The responsible for the research is the principal investigator Mariana Bichuette Cartuliares that together with the Department of Health Research and Hospital of Southern Denmark own the data.

Storage and backup

Data storage, backup and access

Data collection, storage, management and access will be performed in REDCap (Research Electronic Data Capture) in cooperation of ”OPEN, Open Patient data Explorative Network, Odense University Hospital, Region of Southern Denmark”. OPEN’s facilities is concurrent with

applicable legislation on processing of special categories of personal data including health data. OPEN provide any techinical suport.

REDCap is an electronic data capture tools hosted at University of Southern Denmark. It is a secure, web-based software platform designed to support data capture for research studies, providing 1) an intuitive interface for validated data capture; 2) audit trails for tracking data manipulation and export procedures; 3) automated export procedures for seamless data downloads to common statistical packages; and 4) procedures for data integration and interoperability with external sources. [1,2].

1PA Harris, R Taylor, R Thielke, J Payne, N Gonzalez, JG. Conde, Research electronic data capture (REDCap) – A metadata-driven methodology and workflow process for providing translational research informatics support, J Biomed Inform. 2009 Apr;42(2):377-81.

2PA Harris, R Taylor, BL Minor, V Elliott, M Fernandez, L O'Neal, L McLeod, G Delacqua, F Delacqua, J Kirby, SN Duda, REDCap Consortium, The REDCap consortium: Building an international community of software partners, J Biomed Inform. 2019 May 9 [doi: 10.1016/j.jbi.2019.103208]

Additionally, data from the department of microbiology will be stored in the regional close secure system in Sharepoint or hospital logged drive.

Data access and security

Only project assistants that collect data have access to the database via RedCap. Projects assistants have a personal code to entering the system. The data is consequently locked by the project investigator after quality control of each patient is performed.

Share point data is only administered by the principal investigator.

Selection and preservation

Data preservation

Sputum samples will be destroyed after culture results but the results will be reported in the patient medical journal as routinely.

Do-fil format from STATA and R analysis will be stored by OPEN.

According to Danish national law from 2014, other research data related to the study must be preserved in at least five years after the end of the study:

https://ufm.dk/publikationer/2014/the-danish-code-of-conduct-for-research-integrity

The Danish data protection accepted the data from this study (20/41767) to be preserved until 2027.

Long-term preservation of the data

The data will not be preserved in long term.

Data sharing

Data sharing

Due to Danish laws on personal data, data cannot be shared publicly:

https://www.datatilsynet.dk/english/legislation

To request these data, please contact the responsible investigator or corresponding author for more information.

Zenodo will be considered to achieve an increased transparency and an open access to our data to increase the possibility of study reproducibility.

Results will be anonymized before shared.

Responsibilities and resources

Data responsability

The responsible of the data is the investigator and responsible of the study Mariana Bichuette Cartuliares in cooperation to Department of Health Research at University of Southern Denmark and Hospital of Southern Denmark.

The investigator of the study Mariana Bichuette Cartuliares with close cooperation with an independent data manager from OPEN who is responsible for the data control and security will perform data management.

Responsibilities are shared with project assistants and microbiologists in the reporting of the data collection and to assure data quality.

OPEN's data manager will be responsible for the data storage and backup in RedCap. The responsible for the study and OPEN will be responsible for data preservation in five years.

The responsible of the study will have responsibility for data sharing if required.

Resources

Project assistants that collect data will be trained to consistent data collection and they will be available during all study period until the last patient is included in the study.

The chief microbiologist will be in charge to train laborants in gram stain analysis of the sputum.

A statistician from the Hospital of Southern Denmark, Region of Southern Denmark, will perfom data analyses.

OPEN, Odense University Hospital, Region of Southern Denmark will deliver facilities included software and a data manager to supervise the data.

Founding

The University of Southern Denmark and the Hospital of Southern Denmark financially found the study.
