# Supplementary material for: Effectiveness of expiratory technique and induced sputum in obtaining good quality sputum from patients acutely hospitalized with suspected lower respiratory tract infection: a statistical analysis plan for a randomized controlled trial
Source: Trials. 2021 Oct 2;22:675. doi: 10.1186/s13063-021-05639-1 (PMC8487344; doi:10.1186/s13063-021-05639-1)
Supplement: Supplementary file 5 — Additional file 5. Informed consent. [file 13063_2021_5639_MOESM5_ESM.pdf]

Informeret samtykke til at deltage i et sundhedsvidenskabeligt projekt

Projektets titel: Optimal prøvetagning af sekret fra nedre luftveje

Erklæring fra forsøgspersonen:

Jeg har fået skriftlig og mundtlig information, og jeg ved nok om formål, metode, fordele og ulemper til at sige ja til at deltage.

Jeg ved, at det er frivilligt at deltage, og jeg altid kan trække mit samtykke tilbage uden at miste mine nuværende eller fremtidige rettigheder til behandling.

Jeg giver hermed samtykke til at deltage i projektet og har fået en kopi af den skriftlige information om projektet til eget brug.

Projektdeltagers navn:____

Dato: _____________________ Underskrift: ____________________________________

Deltagerinformation om deltagelse i et videnskabeligt projekt, der handler om at finde den bedste metode til at få en sekretprøve fra nedre luftveje ved mistanke om luftvejsinfektion

Projektets titel: Optimal prøvetagning af sekret fra nedre luftveje

Vi vil spørge dig, om du vil deltage i et sundhedsvidenskabeligt forskningsprojekt, hvor Akutmodtagelsen i Aabenraa og Sønderborg samarbejder med Klinisk Mikrobiologisk Afdeling for at finde den bedste metode til at opnå en prøve fra nedre luftveje. Det vil hjælpe lægen til at finde ud af, hvad der er skyld i dine lungesymptomer samt hvilken behandling, der er mest effektiv og skånsom for dig.

Før du beslutter, om du vil deltage i projektet, skal du fuldt ud forstå, hvad det går ud på, og hvorfor vi gennemfører det. Vi vil derfor bede dig om at læse denne deltagerinformation grundigt.

Indhold

Projektets titel: Optimal prøvetagning af sekret fra nedre luftveje 1
1) Hypotese og formål med forsøget 2
2) Plan for projektet 2
3) Deltagelse i forskningsprojektet 3
4) Bivirkninger, risici og ulemper 4
5) Nytte 5
6) Prøvesvar 5
7) Udelukkelse fra og afbrydelse af forsøg 5
8) Initiativtager til projektet 5
9) Økonomisk støtte til projektet 6
10) Adgang til projekts resultater 6
11) Kontaktoplysninger 7
Bilag 1: Forsøgspersoners rettigheder i et sundhedsvidenskabeligt forskningsprojekt 7

1) Hypotese og formål med forsøget

I dag tages sekretprøver fra den nederste del af luftrøret via trakealsugning, med et kateter (en tynd slange). Vores hypotese er, at ved at forbedre hosteteknikken vil patienten selv kunne hoste en sekretprøve op af lige så høj kvalitet som ved sugning.

Formålet med projektet er at sammenligne trakealsugning med en forbedret hosteteknik kombineret med inhalation af saltvand (0.9%) til prøvetagning af sekret fra nedre luftveje for at finde den mest optimale måde, så vi hurtigt kan finde ud af hvilken type luftvejsinfektion, du har, og dermed give dig den bedste behandling.

Projektet vil undersøge følgende:

- Hvilken metode er den bedste til at opnå sekret fra nedre luftveje i relation til egnede prøver
- Hvilken mikrobiologisk metode er bedst til at identificere bakterierne i sekretprøven
- Undersøge om der er bivirkning relateret til de to afprøvede metoder

2) Plan for projektet

Projektet foregår på Sygehus Sønderjylland, hvor i alt 260 patienter som indlægges på Fælles akutmodtagelsen, Aabenraa og medicinsk modtagelse samt lungesygdomme afsnit, Sønderborg inviteres i at deltage.

Personalet på afdelingen vil i forbindelse med din indlæggelse opsøge og informere dig om projektet. Du er velkommen til at have et familiemedlem, en ven eller en bekendt med til samtalen. Da det er vigtigt at indhente sekret fra de nedre luftveje inden for en halv time, beder vi dig om at tilkendegive din beslutning inden for det tidsrum.

3) Deltagelse i forskningsprojektet

Hvis du indvilliger i at deltage i projektet, vil du efter lodtrækning tilfældigt blive tildelt en af to metoder til at skaffe en sekretprøve fra den nedre del af dine luftveje:

- *Metode 1:* Normal procedure på afdelingen, dvs. vores standart undersøgelse, hvor afdelingens personale vil suge sekret op fra nedre luftveje vha. et kateter (tynd slange).
- *Metode 2:* Du hoster selv en sekretprøve op. Du vil modtage instruktion om, hvordan du selv kan hoste en prøve op. Du skal prøve at hoste en prøve op både før og efter indånding af fysiologisk saltvand (0,9%), som gives med en inhalationsmaske. Lykkes det ikke at hoste en prøve op, tages prøven ved standardprocedure, dvs. trakealsugning.

Din sekretprøve vil blive sendt til undersøgelse på Klinisk Mikrobiologisk Afdeling, og vil efter undersøgelsen blive smidt ud. Resultatet af din prøve bliver registreret i din patientjournal.
Vi vil også stille dig nogle spørgsmål om din sygdomme, tidligere indlæggelser samt tidligere og nuværende behandling. Desuden beder vi

om adgang til din elektroniske patientjournal, så vi kan følge op på analyseresultatet af din sekretprøve, have adgang til undersøgelsesresultater som f.eks. blodprøvesvar og røntgenbeskrivelsen under indlæggelsen samt hvilken behandling du har modtaget op til og efter indlæggelsen på akutafdelingen.

Efter indsamlingen af oplysningerne fra dine patientjournal, vil dine persondata blive fjernet, og du vil indgå i projektet i pseudonymiseret form, således at dit personnummer vil blive erstattet af en kode.

4) Bivirkninger, risici og ulemper

Der er ingen risiko, ulemper eller bivirkninger ved at selv at hoste en prøve op og heller ikke efter inhalation af fysiologisk saltvand med en inhalationsmaske. Der kan være bivirkninger ved den konventionelle metode, som er sugning, hvor blødning fra de nedre luftveje kan forekomme, om end det er sjældent og normalt kortvarigt. Begge metoder kan dog opleves som uvante, og særligt ved sugning kan der være kortvarigt ubehag.

Både sugning og saltvandsinhalation er velkendte og almindeligt anvendte procedurer, som vi har stor erfaring med og kendskab til. Der kan dog være risici ved undersøgelserne, som vi endnu ikke kender. Vi beder dig derfor om at fortælle, hvis du oplever problemer i forbindelse med prøvetagningen. Hvis vi opdager bivirkninger, som vi ikke allerede har

fortalt dig om, vil du naturligvis blive orienteret med det samme, og du vil skulle tage stilling til, om du ønsker at fortsætte med prøvetagning.

5) Nytte

Projektet er vigtigt for, at vi kan optimere den metode, vi bruger til at skaffe sekret fra nedre luftveje, så vi hurtigt kan vælge den bedste behandling. Resultatet vil have en afgørende betydning for praksis på akutmodtagelserne og vil bidrage til at sikre optimal behandling, reducere bivirkninger, målrette antibiotikaforbruget, og dermed reducere antibiotikaresistens.

For dig personligt vil projektdeltagelse have betydning for hvilken metode der bliver brugt til at ophente sekret fra dine nedre luftveje. Projektdeltagelse vil ikke have betydning for din videre behandling.

6) Prøvesvar

Ønsker du svar på dine prøver, kan du se det på www.sundhed.dk.

7) Udelukkelse fra og afbrydelse af forsøg

Du vil udgå af projektet, hvis den mikrobiologiske analyse mislykkes eller hvis prøven forsvinder inden analysen iværksættes.

8) Initiativtager til projektet

Projektet er primært udarbejdet i samarbejde mellem Fælles Akutmodtagelsen og Klinisk Mikrobiologiske Afdeling på Sygehus Sønderjylland. Projektet er forankret i Akutforskningsenheden under Institut for Regional Sundhedsforskning på Syddansk Universitet.

9) Økonomisk støtte til projektet

Projektet har fået økonomisk støttet i form af et års ph.d. stipendiat fra Syddansk Universitet (331.000 kr.) og støtte fra Sygehus Sønderjylland til aflønning af personale (25.000 kr.) og mirkobiologiske analyser (8.000 kr.) samt til videnskabeligt publikation (15.000 kr.). Forskeren har ingen økonomisk tilknytning til støttegiveren eller andre interessenter i forsøget.

10) Adgang til projekts resultater

Projektets samlede resultater vil i foråret 2021 blive offentliggjort i et videnskabeligt tidsskrift samt på sygehusets hjemmesiden. Det sikres, at ingen deltagere kan genkendes i det, som offentliggøres. Har du interesse i at blive kontaktet direkte, når forsøgets resultater offentliggøres, kan du sende en mail til nedenstående kontakt.

Vi håber, at du med denne information har fået tilstrækkeligt indblik i, hvad det vil sige at deltage i projektet, og at du føler dig rustet til at tage beslutningen om din eventuelle deltagelse. Information om dine rettigheder er vedlagt denne deltagerinformation sidst i dokumentet (Bilag 1).

Hvis du beslutter dig for at deltage i projektet, vil vi bede dig om at underskrive samtykkeerklæringen. Det er frivilligt at deltage i projektet, og du kan når som helst og uden at give en grund trække dit samtykke tilbage. Det vil ikke få konsekvenser for den videre behandling.

11) Kontaktoplysninger

Yderligere oplysninger kan fås ved henvendelse til nedenstående

Mariana Bichuette Cartuliares

Mariana.cartuliares@rsyd.dk

Ph.d. studerende

Fælles Akutmodtagelsen, Sygehus Sønderjylland

Kresten Philipsens Vej 15 - 6200 Aabenraa

Tlf.: 7997 0000

Bilag 1: Forsøgspersoners rettigheder i et sundhedsvidenskabeligt forskningsprojekt

Som deltager i et sundhedsvidenskabeligt forskningsprojekt skal du vide, at:

• din deltagelse i forskningsprojektet er helt frivillig og kun kan ske efter, at du har fået både skriftlig og mundtlig information om forskningsprojektet og underskrevet samtykkeerklæringen.

• du til enhver tid mundtligt, skriftligt eller ved anden klar tilkendegivelse kan trække dit samtykke til deltagelse tilbage og udtræde af forskningsprojektet. Såfremt du trækker dit samtykke tilbage påvirker dette ikke din ret til nuværende eller fremtidig behandling eller andre rettigheder, som du måtte have.

• du har ret til at tage et familiemedlem, en ven eller en bekendt med til informationssamtalen.

• du har ret til betænkningstid, før du underskriver samtykkeerklæringen.

• oplysninger om dine helbredsforhold, øvrige rent private forhold og andre fortrolige oplysninger om dig, som fremkommer i forbindelse med forskningsprojektet, er omfattet af tavshedspligt.

• behandling af oplysninger om dig, herunder oplysninger i dine blodprøver og væv, sker efter reglerne i databeskyttelsesforordningen, databeskyttelsesloven samt sundhedsloven. Den dataansvarlige i forsøget skal orientere dig nærmere om dine rettigheder efter databeskyttelsesreglerne.

• der er mulighed for at få aktindsigt i forsøgsprotokoller efter offentlighedslovens bestemmelser. Det vil sige, at du kan få adgang til at se alle papirer vedrørende forsøgets tilrettelæggelse, bortset fra de dele, som indeholder forretningshemmeligheder eller fortrolige oplysninger om andre.
• der er mulighed for at klage og få erstatning efter reglerne i lov om klage- og erstatningsadgang inden for sundhedsvæsenet. Hvis der under forsøget skulle opstå en skade kan du henvende dig til Patienterstatningen, se nærmere på www.patienterstatningen.dk

**De Videnskabsetiske Komiteer for Region Hovedstaden (6 komiteer)**<br>Tlf.: +45 38 66 63 95<br>E-mail: vek@regionh.dk<br>Hjemmeside:<br>https://www.regionh.dk/til-fagfolk/Forskning-og-innovation/Kliniske-test-og-forsoeg/Sider/De-Videnskabsetiske-Komitéer.aspx	**De Videnskabsetiske Komiteer for Region Syddanmark (2 komiteer)**<br>Tlf.: + 45 76 63 82 21<br>E-mail: komite@rsyd.dk<br>Hjemmeside:<br>https://komite.regionsyddanmark.dk/wm258128	**Den Videnskabsetiske Komité for Region Nordjylland** Tlf.: +45 97 64 84 40<br>E-mail: vek@rn.dk<br>Hjemmeside:<br>http://www.rn.dk/vek
**Den Videnskabsetiske Komité for Region Sjælland**<br>Tlf.: +45 93 56 60 00<br>E-mail: RVK-sjaelland@regionsjaelland.dk<br>Hjemmeside:<br>https://www.regionsjaelland.dk/sundhed/forskning/forfagfolk/videnskabsetisk-komite/Sider/default.aspx	**De Videnskabsetiske Komiteer for Region Midtjylland (2 komiteer)**<br>Tlf.: +45 78 41 01 83 / +45 78 41 01 82 / +45 78 41 01 81<br>E-mail: komite@rm.dk<br>Hjemmeside:<br>http://www.komite.rm.dk	**National Videnskabsetisk Komité**<br>Tlf.: +45 72 21 68 55<br>E-mail: kontakt@nvk.dk<br>Hjemmeside: http://www.nvk.dk

Dette tillæg er udarbejdet af det Videnskabsetiske komitésystem og kan vedhæftes den skriftlige information om det sundhedsvidenskabelige forskningsprojekt. Spørgsmål til et konkret projekt skal rettes til projektets forsøgsansvarlige. Generelle spørgsmål til forsøgspersoners rettigheder kan rettes til den komité, som har godkendt projektet.
Revideret 21. september 2019
