# Supplementary material for: Effectiveness of expiratory technique and induced sputum in obtaining good quality sputum from patients acutely hospitalized with suspected lower respiratory tract infection: a statistical analysis plan for a randomized controlled trial
Source: Trials. 2021 Oct 2;22:675. doi: 10.1186/s13063-021-05639-1 (PMC8487344; doi:10.1186/s13063-021-05639-1)
Supplement: Supplementary file 6 — Additional file 6. SAP guideline. [file 13063_2021_5639_MOESM6_ESM.pdf]

JAMA | Special Communication

Guidelines for the Content of Statistical Analysis Plans in Clinical Trials

Carrol Gamble, PhD; Ashma Krishan, BSc; Deborah Stocken, PhD; Steff Lewis, PhD; Edmund Juszczak, MSc; Caroline Doré, BSc; Paula R. Williamson, PhD; Douglas G. Altman, DSc; Alan Montgomery, PhD; Pilar Lim, PhD; Jesse Berlin, ScD; Stephen Senn, PhD; Simon Day, PhD; Yolanda Barbachano, PhD; Elizabeth Loder, MD, MPH

IMPORTANCE While guidance on statistical principles for clinical trials exists, there is an absence of guidance covering the required content of statistical analysis plans (SAPs) to support transparency and reproducibility.

OBJECTIVE To develop recommendations for a minimum set of items that should be addressed in SAPs for clinical trials, developed with input from statisticians, previous guideline authors, journal editors, regulators, and funders.

DESIGN Funders and regulators (n = 39) of randomized trials were contacted and the literature was searched to identify existing guidance; a survey of current practice was conducted across the network of UK Clinical Research Collaboration–registered trial units (n = 46, 1 unit had 2 responders) and a Delphi survey (n = 73 invited participants) was conducted to establish consensus on SAPs. The Delphi survey was sent to statisticians in trial units who completed the survey of current practice (n = 46), CONSORT (Consolidated Standards of Reporting Trials) and SPIRIT (Standard Protocol Items: Recommendations for Interventional Trials) guideline authors (n = 16), pharmaceutical industry statisticians (n = 3), journal editors (n = 9), and regulators (n = 2) (3 participants were included in 2 groups each), culminating in a consensus meeting attended by experts (N = 12) with representatives from each group. The guidance subsequently underwent critical review by statisticians from the surveyed trial units and members of the expert panel of the consensus meeting (N = 51), followed by piloting of the guidance document in the SAPs of 5 trials.

FINDINGS No existing guidance was identified. The registered trials unit survey (46 responses) highlighted diversity in current practice and confirmed support for developing guidance. The Delphi survey (54 of 73, 74% participants completing both rounds) reached consensus on 42% (n = 46) of 110 items. The expert panel (N = 12) agreed that 63 items should be included in the guidance, with an additional 17 items identified as important but may be referenced elsewhere. Following critical review and piloting, some overlapping items were combined, leaving 55 items.

CONCLUSIONS AND RELEVANCE Recommendations are provided for a minimum set of items that should be addressed and included in SAPs for clinical trials. Trial registration, protocols, and statistical analysis plans are critically important in ensuring appropriate reporting of clinical trials.

JAMA. 2017;318(23):2337-2343. doi:10.1001/jama.2017.18556

Editorial page 2301

Supplemental content

CME Quiz at jamanetwork.com/learning and CME Questions page 2348

Author Affiliations: Author affiliations are listed at the end of this article.

Corresponding Author: Carrol Gamble, PhD, Biostatistics Department, Block F Waterhouse Building, 1-5 Brownlow St, University of Liverpool, Liverpool L69 3GL, England (c.gamble@liverpool.ac.uk).

© 2017 American Medical Association. All rights reserved.

Downloaded From: by a Copenhagen University Library User on 12/21/2017

Transparency has been described as a fundamental value of society and initiatives to increase transparency in relation to clinical trial data have been launched.[1] Given the influence of statistical decisions on trial conclusions, well-documented and transparent statistical conduct is essential. This is relevant given concerns regarding research reproducibility.[2]

The contribution of the statistician to the design and analysis of clinical trials is acknowledged to be essential.[3] Guidance on statistical principles for clinical trials (International Conference for Harmonisation of Technical Requirements for Pharmaceuticals for Human Use [ICH] E9)[4] state that "the principal features of the eventual statistical analysis of the data should be described in the statistical section of the protocol." However, ICH E9[4] and SPIRIT (Standard Protocol Items: Recommendations for Interventional Trials)[5] guidelines refer to a separate statistical analysis plan (SAP). The level of detail appropriate for a SAP exceeds that of a protocol. According to ICH E9,[4] a SAP "contains a more technical and detailed elaboration of the principal features of the analysis described in the protocol, and includes detailed procedures for executing the statistical analysis of the primary and secondary variables and other data." While guidance exists on the content of clinical trial protocols[5] and reporting standards for clinical trials,[6] both of which require a summary of the statistical analyses, there is no guidance on SAP content. Consequently, there is marked variation in practice.

This Special Communication provides recommendations for a minimum set of items that should be addressed and describes the methods used to develop this list. The recommendations are intended to aid the drafting of SAPs for clinical trials and improve their completeness.

Methods

The need to develop guidance on SAPs was raised during discussion by statisticians attending a UK Clinical Research Collaboration (UKCRC) Registered CTU (Clinical Trials Unit) Statisticians' Operational Group meeting in November 2012. This group included 46 senior statisticians, each representing their CTU within the network. This wider group was engaged throughout the development process as well as user-testing and piloting. The members of the CTU network, based in the United Kingdom, conduct clinical trials funded by governmental agencies, foundations, and pharmaceutical companies under the remit of the European Medicines Agency, the UK Medicines and Healthcare Products Regulatory Agency (MHRA), and the US Food and Drug Administration. An application for funding was developed and submitted to the Medical Research Council Network of Hubs for Trials Methodology Research in December 2013 and the project started in May 2014. The SAP guidance document was developed with the primary intention of being applicable to the final analyses of later-phase randomized clinical trials addressing the minimum recommended content of a SAP within the context of the following assumptions:

1. The SAP is not a standalone document and should be read in conjunction with the clinical trial protocol;
2. The clinical trial protocol should be consistent with the principles of the SPIRIT 2013 Statement[5]; and
3. The SAP is to be applied to a clean or validated data set for analysis.

This guidance document summarizes the findings of a comprehensive search to identify existing SAP guidance; a survey of current practice of statisticians within UKCRC-registered CTUs; and a Delphi survey to establish consensus. Consistent with advice received from the Central Office of Research Ethics, the UK Health Research Authority Decision Tool[7] indicated ethical approval was not required for the surveys and consent to take part was indicated by survey participation.

Identification of Guidance

Major randomized clinical trial funding bodies and regulators were identified from responses to a previous survey,[8] which had generated a list of funders actively supporting clinical trials across at least 2 CTUs within the last 5 years. The full list is contained in eTable 1 in the Supplement and includes the European and Developing Countries Clinical Trials Partnership, FP7 Health Research, Medical Council of Canada, National Cancer Institute of Canada Clinical Trials Group, European Organisation for Research and Treatment for Cancer, National Institutes of Health, and the National Institute for Health Research. The list, which was reviewed by the project team (May 2014), was extended to include regulators (US Food and Drug Administration, European Medicines Agency, and MHRA).

All funders and regulators were contacted by email (June 2014). If a response was not received, up to 2 further reminder emails were sent. If no response was received, the organization was contacted by telephone and the study team discussed whether alternative contacts within the organization could be approached to participate.

Journals were contacted in parallel to funders and regulators, and included *JAMA*, *BMJ*, the *New England Journal of Medicine*, and the *Lancet* as the leading medical journals publishing clinical trials. Journals identified via a PubMed search (June 2014) publishing SAPs as standalone publications were also contacted (*Trials*, *Critical Care and Resuscitation*, and *International Journal of Stroke*). The goal was to identify whether the journals had any internal guidance or recommendations on SAPs, if they followed any externally available guidance on SAPs, whether and how they used SAPs within the peer-review process, and any policies on the publications of SAPs. Each journal website was searched for information relating to SAPs within their support for authors and reviewers prior to contacting a journal editor.

Survey of Current Practice

The aim of the survey was to identify current practice and opinions about SAPs. A list of the 45 registered CTUs was accessed from the UKCRC website (June 2014). One CTU reported being split across 2 sites, with each using separate standard operating procedures, and requested that each site complete the survey separately. The survey was developed by A.K., C.G., and D.S. and adapted in response to comments from the project team. To reduce the number of survey questions, copies of standard operational procedures for SAPs and templates or examples of SAPs were also requested. In addition, the survey was piloted during July 2014 by statisticians from the CTUs of the study project team prior to distribution.

A senior statistician at each CTU, identified as the network's nominated statistics contact, was asked to complete the survey to reflect practices and majority opinion within the statistician's CTU

© 2017 American Medical Association. All rights reserved.

Downloaded From: by a Copenhagen University Library User on 12/21/2017

(August 2014). For networks in which there was no nominated statistics contact, the survey was sent to the CTU director who was asked to delegate completion on behalf of the unit. Two reminder emails were sent to encourage responses. Survey completion was highlighted at network events at which nonresponders were approached to discuss completion. A copy of the survey and the participating CTUs is provided in eAppendix 1 in the Supplement.

The Delphi Survey

Participants

The aim of the Delphi survey was to establish consensus among a broad range of stakeholders. The initial list of participants was sent to the project team for review and amendment (January 2015). The UKCRC-registered CTU participants were identified from the survey of current practice (n = 46). CONSORT and SPIRIT guideline authors were identified from relevant publications and websites (n = 16). Pharmaceutical industry contributors were selected from recommendations from the project team and aimed to have both industry and academic experience (n = 5). The journal editors contacted to identify existing guidance were also contacted to participate in the Delphi survey (n = 7). Regulators from the European Medicines Agency and the MHRA were included (n = 2). Contacts with the US Food and Drug Administration were unsuccessful in identifying a participant for the Delphi survey.

Delphi Contents

A comprehensive list of items that should or could be included within a SAP was derived after reviewing suggested guidance identified from contacting funders and regulators, considering the responses to the survey of current practice, and reviewing copies of standard operational procedures for SAPs and examples of SAPs provided with the survey responses or identified in the literature search. Items were listed individually but grouped under relevant domains.

The list was reviewed by the project team for completeness, comprehension, and suitability of the domains (January 2015). The Delphi survey was completed during February 2015, with each round lasting 2 weeks. During round 1, Delphi participants could suggest additional items for inclusion in round 2. Round 2 included all items from round 1 as well as the additional items suggested by participants. Suggestions were reviewed by the project team and checked for duplication prior to inclusion in round 2.

Scoring Process

Participants were asked to score the importance of each item when writing, following, or reviewing a SAP. The scale was presented with 1 to 3 labeled "not important," 4 to 6 labeled "important but not critical," and 7 to 9 labeled "critical."[9]

All individual participants who completed round 1 were emailed and asked to complete round 2. In round 2, for each item, participants were presented with the number and percentage of participants who chose each score. Participants were shown their score from round 1 and provided with an option to revise their score for each of the items or keep it the same as their score in round 1.

Consensus Meeting

The definition of consensus was predefined and is presented in eTable 2 in the Supplement. Items were determined to be in (consensus-in) if 70% or more of participants scored the item as critical and less than 15% of participants scored the item as not important. Items were deleted (consensus-out) if 70% or more of participants scored it as not important and less than 15% of participants scored it as critical.

Following round 2 of the Delphi process, a consensus meeting was held (March 2015) with expert representation from each group: CTU senior statisticians, regulators (MHRA), statisticians in the pharmaceutical industry, and journal editors. The 12 expert panel members are listed in eTable 3 in the Supplement.

All items included in the Delphi survey were reviewed at the consensus meeting. Items on which consensus had been reached were highlighted but not discussed further. The expert panel members were asked to discuss each item for which consensus had not been reached and, following discussion, to make a recommendation regarding its inclusion with consensus-in items within the minimum set of items that should be addressed and included in SAPs for clinical trials.

Critical Review and Piloting

The aim of the critical review and piloting was to ensure the guidance produced was fit for purpose, appropriate to the needs of statisticians authoring and implementing SAPs, and to identify any items requiring clarification. The first draft of the guidance underwent critical review by attendees at the UKCRC Registered CTU Statisticians' Operational Group meeting in April 2015. Meeting attendees were able to provide additional comments based on further discussions with the statistics team within their CTU until September 2015. Following incorporation of comments, the guidance was sent to the expert panel involved in the Delphi consensus meeting prior to being piloted by senior statisticians across 5 trials in January 2016.

Results

Of the 39 funding bodies or regulators that were contacted and asked about their requirements or guidance for SAPs, 28 responded (72%). Four responders referred to ICH E9,[4] 3 to the UK Medical Research Council website or ICH Good Clinical Practice guidance,[3] and 21 indicated an absence of guidance or recommendations relevant to SAPs. A comprehensive search of the literature and references of published SAPs did not identify any publications relevant to the content of SAPs.

The survey to establish current practice was distributed by email to each of the 45 UKCRC-registered CTUs (46 respondents), with a 100% response rate. Responses demonstrated variability in current practice around the processes of producing SAPs and their content. The production of guidance on SAP content was supported by 85% (n = 39) of responders.

Of the 73 invited participants in the Delphi process, 56 (77%) completed round 1 and 54 (73%), round 2. Those completing round 2 included CTU statisticians (40/46; 87%), editors (3/7; 43%), guideline authors (8/16; 50%), industry (5/5; 100%), and a regulator (1/2; 50%) (3 responders contributed to 2 groups each). Thirty percent of the responders were from outside the United Kingdom and included Canada, Germany, Ireland, Denmark, Australia, and the United States.

Round 1 contained 89 items, consensus for items to remain in was reached on 28 items, and an additional 21 items were

© 2017 American Medical Association. All rights reserved.

Downloaded From: by a Copenhagen University Library User on 12/21/2017

Table. SAP Guidance Document: Recommended Items to Address in a Clinical Trial SAP[a]

Section/Item	Index	Description
**Section 1: Administrative Information**		
Title and trial registration	1a	Descriptive title that matches the protocol, with SAP either as a forerunner or subtitle, and trial acronym (if applicable)
	1b	Trial registration number
SAP version	2	SAP version number with dates
Protocol version	3	Reference to version of protocol being used
SAP revisions	4a	SAP revision history
	4b	Justification for each SAP revision
	4c	Timing of SAP revisions in relation to interim analyses, etc
Roles and responsibility	5	Names, affiliations, and roles of SAP contributors
Signatures of:	6a	Person writing the SAP
	6b	Senior statistician responsible
	6c	Chief investigator/clinical lead
**Section 2: Introduction**		
Background and rationale	7	Synopsis of trial background and rationale including a brief description of research question and brief justification for undertaking the trial
Objectives	8	Description of specific objectives or hypotheses
**Section 3: Study Methods**		
Trial design	9	Brief description of trial design including type of trial (eg, parallel group, multiarm, crossover, factorial) and allocation ratio and may include brief description of interventions
Randomization	10	Randomization details, eg, whether any minimization or stratification occurred (including stratifying factors used or the location of that information if it is not held within the SAP)
Sample size	11	Full sample size calculation or reference to sample size calculation in protocol (instead of replication in SAP)
Framework	12	Superiority, equivalence, or noninferiority hypothesis testing framework, including which comparisons will be presented on this basis
Statistical interim analyses and stopping guidance	13a	Information on interim analyses specifying what interim analyses will be carried out and listing of time points
	13b	Any planned adjustment of the significance level due to interim analysis
	13c	Details of guidelines for stopping the trial early
Timing of final analysis	14	Timing of final analysis, eg, all outcomes analyzed collectively or timing stratified by planned length of follow-up
Timing of outcome assessments	15	Time points at which the outcomes are measured including visit "windows"
**Section 4: Statistical Principles**		
Confidence intervals and *P* values	16	Level of statistical significance
	17	Description and rationale for any adjustment for multiplicity and, if so, detailing how the type 1 error is to be controlled
	18	Confidence intervals to be reported
Adherence and protocol deviations	19a	Definition of adherence to the intervention and how this is assessed including extent of exposure
	19b	Description of how adherence to the intervention will be presented
	19c	Definition of protocol deviations for the trial
	19d	Description of which protocol deviations will be summarized
Analysis populations	20	Definition of analysis populations, eg, intention to treat, per protocol, complete case, safety
**Section 5: Trial Population**		
Screening data	21	Reporting of screening data (if collected) to describe representativeness of trial sample
Eligibility	22	Summary of eligibility criteria
Recruitment	23	Information to be included in the CONSORT flow diagram
Withdrawal/follow-up	24a	Level of withdrawal, eg, from intervention and/or from follow-up
	24b	Timing of withdrawal/lost to follow-up data
	24c	Reasons and details of how withdrawal/lost to follow-up data will be presented
Baseline patient characteristics	25a	List of baseline characteristics to be summarized
	25b	Details of how baseline characteristics will be descriptively summarized

(continued)

© 2017 American Medical Association. All rights reserved.

Downloaded From: by a Copenhagen University Library User on 12/21/2017

Table. SAP Guidance Document: Recommended Items to Address in a Clinical Trial SAP[a] (continued)

Section/Item	Index	Description
**Section 6: Analysis**		
Outcome definitions		List and describe each primary and secondary outcome including details of:
	26a	specification of outcomes and timings. If applicable include the order of importance of primary or key secondary end points (eg, order in which they will be tested)
	26b	specific measurement and units (eg, glucose control, hbA_{1c} [mmol/mol or %])
	26c	any calculation or transformation used to derive the outcome (eg, change from baseline, QoL score, time to event, logarithm, etc)
Analysis methods	27a	what analysis method will be used and how the treatment effects will be presented
	27b	any adjustment for covariates
	27c	methods used for assumptions to be checked for statistical methods
	27d	details of alternative methods to be used if distributional assumptions do not hold, eg, normality, proportional hazards, etc
	27e	any planned sensitivity analyses for each outcome where applicable
	27f	any planned subgroup analyses for each outcome including how subgroups are defined
Missing data	28	Reporting and assumptions/statistical methods to handle missing data (eg, multiple imputation)
Additional analyses	29	Details of any additional statistical analyses required, eg, complier-average causal effect[10] analysis
Harms	30	Sufficient detail on summarizing safety data, eg, information on severity, expectedness, and causality; details of how adverse events are coded or categorized; how adverse event data will be analyzed, ie, grade 3/4 only, incidence case analysis, intervention emergent analysis
Statistical software	31	Details of statistical packages to be used to carry out analyses
References	32a	References to be provided for nonstandard statistical methods
	32b	Reference to Data Management Plan
	32c	Reference to the Trial Master File and Statistical Master File
	32d	Reference to other standard operating procedures or documents to be adhered to

Abbreviations: CONSORT, Consolidated Standards of Reporting Trials; hbA_{1c}, hemoglobin A_{1c}; QoL, quality of life; SAP, statistical analysis plan.

[a] Reproduced with permission from the authors.

suggested by responders. Round 2 contained 110 items (89 prepopulated items from round 1 and the 21 suggested items) and at the end of round 2, consensus was reached that 46 items should remain in with 1 item deleted (consensus-out).

At the end of the consensus meeting, there were 63 items in (consensus-in), 30 items deleted (consensus-out), and 17 items that the expert panel felt are important but do not necessarily need to be included (eTable 4 in the Supplement). These 17 items may be found in other trial documents but the SAP should incorporate references to where details of these items can be found.

The critical review meeting, held in London, was attended by 51 statisticians from 37 CTUs (April 2015). Participants were asked to consider the ordering and clarity of the descriptions of each of the 63 items and to highlight any concerns. To ensure discussion and complete coverage of the items within the meeting, attendees were split into groups, with each group allocated 1 of the 6 sections to review and provide feedback on as a priority. Meeting attendees were also encouraged to discuss the draft guidance with other statisticians within their CTUs and return any additional collective responses. Additional responses were received from 8 CTUs.

Two issues were raised: the first was whether the sample size calculation should be replicated from the protocol in full or referenced and the second was concerning the use of a 2-stage analysis in which the assumptions of the analysis approach are tested and then the analysis determined by whether the assumptions are met or not. The sample size statement was amended to support an individual statistician's preference to replicate or reference the protocol. The issue surrounding the 2-stage analysis was more controversial and in response to discussions, the guidance was amended to ensure that this was highlighted in the discussion of that item. During critical review of the 63 items, some items were found to overlap and were combined, leaving 55. The **Table** displays the essential items and their subitems. There are 6 sections: Title and Trial Registration (11 items/subitems); Introduction (2 items); Study Methods (9 items/subitems); Statistical Principles (8 items/subitems); Trial Population (8 items/subitems); and Analysis (17 items/subitems).

An open request for 5 volunteers to undertake piloting of the recommendations in the guidance document was made at the critical review meeting. Twelve statisticians expressed an interest and were invited to participate; 5 were selected to cover CTUs with varying experience in Wales, England, and Scotland, each of whom applied the guidance document to trials in adults and children, and included pharmaceutical and nonpharmaceutical interventions including devices and physiotherapy. The piloting feedback did not require any changes to the guidance and the comments received supported its content and usability.

An elaboration and explanation of each item is included within eAppendix 2 in the Supplement. Examples are provided to illustrate each item, along with an explanation of the rationale and detailed description of the issues to be addressed. Examples for each item are based on real SAPs either published in journals, provided by responders to the CTU survey, or contained within National Institute for Health Research's Health Technology Assessment monographs.

© 2017 American Medical Association. All rights reserved.

Downloaded From: by a Copenhagen University Library User on 12/21/2017

Discussion

It is important that every clinical trial has a clear and comprehensive SAP to support reproducibility. Leading organizations and funding bodies openly support data sharing as best practice for clinical trials.[11] Such support will undoubtedly increase the availability of data from original research, resulting in an increase of attempts to replicate results. To support the reproducibility of research and allay concerns of misconduct and fraud in clinical research, a clear comprehensive and transparent account of preplanned statistical analyses must be available.[12] The aim of this guidance is to establish the minimum set of essential items required for a SAP for a clinical trial. It is intended to lead to improvements in the integrity of trial conduct and reporting by facilitating critical appraisal, execution, replication, and identification of any deviations from the prespecified methods.

This SAP guidance was developed following established transparent methods and involving a diverse range of stakeholders involved in the design, funding, conduct, review, and publication of clinical trials. Although the guidance was developed with a focus on the regulatory requirements of trials of medicinal products, and in particular later-phase trials, many aspects are transferable to studies of other types of interventions, phases, and designs.

This guidance document does not cover when a SAP should be written, but early authoring of SAPs—before any data have been collected or analyzed—is the best approach. The final opportunity to amend the SAP should be in response to blind review, defined as the checking and assessment of data during the period between trial completion and the breaking of the blind, the act of unveiling each participant's random allocation.[4] Following this point, deviations from the SAP and additional analyses should be clearly indicated as such within all reports and publications.[4] In the United Kingdom, the Health Research Authority has developed a protocol template[13] to improve consistency in the way that the items covered by SPIRIT are included within a protocol and a similar template may be beneficial for SAPs.

This guidance assumes that the SAP is not a standalone document, and therefore, it is not necessary to replicate large portions of the protocol, which should instead be clearly referenced. The SAP should contain a statement that it is consistent with the principal features of the statistical methods described in the protocol or a section detailing which analyses are different to those planned in the protocol and why. Any abbreviations used should be spelled out in full.

SAPs should be made publicly available.[14] A major step toward public availability of SAPs is the requirements of the US National Institutes of Health Final Rule for Clinical Trials Registration and Results Information Submission,[15] which in addition to posting of results within ClinicalTrials.gov also requires posting of the SAP if not contained within the protocol. In the discussion of public comments relating to the Final Rule,[15] it was noted that many of the benefits of the protocol that were cited by commenters were derived from the information regarding the statistical analyses. This represents acknowledgment that SAPs have an important role in reducing the occurrence of, and facilitating the detection of, bias particularly in relation to selective analysis and reporting.[16,17] Some journals, including *JAMA*, require the SAP to be submitted alongside the report of a clinical trial for use within the peer-review process. The SAP may be made available as supplementary material or published as a standalone article. While this is encouraging, and increases public availability of SAPs, there is no guidance on how the SAP should be used or evaluated. Similar to protocols, the ability of a SAP to provide transparency is dependent on its content.

Any guidance needs to be responsive to relevant information from future projects and initiatives, as well as changes in legislation. Key initiatives that may influence SAP content include the addendum to ICH E9 on estimands and sensitivity analyses,[18] data-sharing initiatives,[19] and mandatory requirements to post clinical trial results in the European Clinical Trials Database and ClinicalTrials.gov.[15,20,21] Future revisions of this document will be made available periodically and extensions to other study designs, including observational studies[22] and studies with adaptive designs and Bayesian analyses, should be considered.

Conclusions

Recommendations are provided for a minimum set of items that should be addressed and included in SAPs for clinical trials. Trial registration, protocols, and statistical analysis plans are critically important in ensuring appropriate reporting of clinical trials.

ARTICLE INFORMATION

Accepted for Publication: November 7, 2017.

Author Affiliations: Biostatistics Department, University of Liverpool, Liverpool, England (Gamble, Williamson); Clinical Trials Research Centre, University of Liverpool, Liverpool, England (Krishan); Newcastle University, Newcastle, England (Stocken); Currently with Leeds Institute of Clinical Trials Research, University of Leeds, Leeds, England (Stocken); Edinburgh University, Edinburgh, Scotland (Lewis); University of Oxford, Oxford, England (Juszczak); UCL Comprehensive Clinical Trials Unit, London, England (Doré); Centre for Statistics in Medicine, University of Oxford, Oxford, England (Altman); University of Nottingham, Nottingham, England (Montgomery); Janssen Research & Development LLC, Raritan, New Jersey (Lim); Johnson & Johnson, Titusville, New Jersey (Berlin); Luxembourg Institute of Health, Strassen, Luxembourg (Senn); Clinical Trials Consulting & Training Limited, Buckingham, England (Day); Medicines and Healthcare Products Regulatory Agency, London, England (Barbachano); *BMJ*, London, England (Loder).

Author Contributions: Dr Gamble and Ms Krishan had full access to all of the data in the study and take responsibility for the integrity of the data and the accuracy of the data analysis.
Concept and design: Gamble, Stocken, Lewis, Dore, Williamson, Montgomery, Lim, Berlin, Senn.
Acquisition, analysis, or interpretation of data: Gamble, Krishan, Stocken, Juszczak, Dore, Williamson, Altman, Montgomery, Lim, Day, Barbachano, Loder.
Drafting of the manuscript: Gamble, Krishan, Stocken, Dore, Altman, Lim.
Critical revision of the manuscript for important intellectual content: Gamble, Stocken, Lewis, Juszczak, Dore, Williamson, Montgomery, Lim, Berlin, Senn, Day, Barbachano, Loder.
Statistical analysis: Gamble, Krishan, Stocken, Williamson, Senn.
Obtained funding: Gamble, Stocken, Lewis, Juszczak, Dore, Williamson, Montgomery.
Administrative, technical, or material support: Williamson, Lim.
Supervision: Gamble, Stocken.

Conflict of Interest Disclosures: All authors have completed and submitted the ICMJE Form for Disclosure of Potential Conflicts of Interest. Dr Berlin is a full-time employee of Johnson & Johnson. Dr Loder is head of research for *BMJ*. No other disclosures were reported.

© 2017 American Medical Association. All rights reserved.

Downloaded From: by a Copenhagen University Library User on 12/21/2017

Funding/Support: This work was funded by grant MR/L004933/1-R44 from the UK Medical Research Council Network of Hubs for Trials Methodology Research and supported and endorsed by the UK Clinical Research Collaboration Registered Clinical Trials Unit Network.

Role of the Funder/Sponsor: The funders/sponsors had no role in the design and conduct of the study; collection, management, analysis and interpretation of the data; preparation review, or approval of the manuscript; and decision to submit the manuscript for publication.

REFERENCES

1. Bonini S, Eichler HG, Wathion N, Rasi G. Transparency and the European Medicines Agency: sharing of clinical trial data. *N Engl J Med*. 2014;371(26):2452-2455.

2. Baker M. Is there a reproducibility crisis? a *Nature* survey lifts the lid on how researchers view the crisis rocking science and what they think will help. *Nature*. 2016;533(7604):452-455.

3. International Council for Harmonisation of Technical Requirements for Pharmaceuticals for Human Use. *ICH Harmonised Guideline: Integrated addendum to ICH E6(R1): Guideline for Good Clinical Practice E6(R2)*. London, England: European Medicines Agency; 2016.

4. International Conference on Harmonisation of Technical Requirements for Registration of Pharmaceuticals for Human Use. *ICH Harmonised Tripartite Guideline: Statistical Principles for Clinical Trials E9*. London, England: European Medicines Agency; 1998.

5. Chan A-W, Tetzlaff JM, Gøtzsche PC, et al. SPIRIT 2013 explanation and elaboration: guidance for protocols of clinical trials. *BMJ*. 2013;346:e7586.

6. Moher D, Hopewell S, Schulz KF, et al; Consolidated Standards of Reporting Trials Group. CONSORT 2010 explanation and elaboration: updated guidelines for reporting parallel group randomised trials. *J Clin Epidemiol*. 2010;63(8):e1-e37.

7. UK Health Research Authority. HRA decision tool. http://www.hra.nhs.uk/research-community/before-you-apply/determine-whether-your-study-is-research/. Accessed November 6, 2017.

8. Conroy EJ, Harman NL, Lane JA, et al. Trial steering committees in randomised controlled trials: a survey of registered clinical trials units to establish current practice and experiences. *Clin Trials*. 2015;12(6):664-676.

9. Guyatt GH, Oxman AD, Kunz R, et al. GRADE guidelines, 2: framing the question and deciding on important outcomes. *J Clin Epidemiol*. 2011;64(4):395-400.

10. Dunn G, Maracy M, Tomenson B. Estimating treatment effects from randomized clinical trials with noncompliance and loss to follow-up: the role of instrumental variable methods. *Stat Methods Med Res*. 2005;14(4):369-395.

11. Taichman DB, Sahni P, Pinborg A, et al. Data Sharing statements for clinical trials: a requirement of the International Committee of Medical Journal Editors. secondary data sharing statements for clinical trials. http://www.icmje.org/news-and-editorials/data_sharing_june_2017.pdf. Published June 6, 2017. Accessed November 11, 2017.

12. Herson J. Strategies for dealing with fraud in clinical trials. *Int J Clin Oncol*. 2016;21(1):22-27.

13. Health Research Authority. Protocol guidance and template for use in a Clinical Trial of an Investigational Medicinal Product (CTIMP): secondary protocol guidance and template for use in a Clinical Trial of an Investigational Medicinal Product (CTIMP) 2015. http://www.hra.nhs.uk/about-the-hra/consultations-calls/closed-consultations/protocol-guidance-template-use-clinical-trial-investigational-medicinal-product-ctimp-consultation-use/. Accessed October 13, 2017.

14. Finfer S, Bellomo R. Why publish statistical analysis plans? *Crit Care Resusc*. 2009;11(1):5-6.

15. National Institutes of Health, Department of Health and Human Services. Final rule for clinical trials registration and results information submission: 42 CFR Part 11. https://www.federalregister.gov/documents/2016/09/21/2016-22129/clinical-trials-registration-and-results-information-submission. Published September 21, 2016. Accessed October 13, 2017.

16. Dwan K, Altman DG, Clarke M, et al. Evidence for the selective reporting of analyses and discrepancies in clinical trials: a systematic review of cohort studies of clinical trials. *PLoS Med*. 2014;11(6):e1001666.

17. Kirkham JJ, Dwan KM, Altman DG, et al. The impact of outcome reporting bias in randomised controlled trials on a cohort of systematic reviews. *BMJ*. 2010;340:c365.

18. International Conference on Harmonisation of Technical Requirements for Registration of Pharmaceuticals for Human Use. Final concept paper: E9(R1): addendum to statistical principles for clinical trials on choosing appropriate estimands and defining sensitivity analyses in clinical trials. http://www.ich.org/fileadmin/Public_Web_Site/ICH_Products/Guidelines/Efficacy/E9/E9_R1_Final_Concept_Paper_October_23_2014.pdf. Accessed October 13, 2017.

19. Smith CT, Hopkins C, Sydes M, et al. How should individual participant data (IPD) from publicly funded clinical trials be shared? *BMC Med*. 2015;13(1):1.

20. European Commission. Commission guideline: guidance on posting and publication of result-related information on clinical trials in relation to the implementation of article 57(2) of regulation (EC) No. 726/2004 and article 41(2) of regulation (EC) No. 1901/2006. http://ec.europa.eu/health/files/eudralex/vol-10/2012_302-03/2012_302-03_en.pdf. Accessed October 13, 2017.

21. European Commission. Technical guidance on the format of the data fields of result-related information on clinical trials submitted in accordance with article 57(2) of regulation (EC) No. 726/2004 and article 41(2) of regulation (EC) No. 1901/2006. http://ec.europa.eu/health/files/eudralex/vol-10/2013_01_22_tg_en.pdf. Accessed October 13, 2017.

22. Thomas L, Peterson ED. The value of statistical analysis plans in observational research: defining high-quality research from the start. *JAMA*. 2012;308(8):773-774.

© 2017 American Medical Association. All rights reserved.

Downloaded From: by a Copenhagen University Library User on 12/21/2017
